# Assessing the impact of an educational intervention program on sexual abstinence based on the health belief model amongst adolescent girls in Northern Ghana, a cluster randomised control trial

**DOI:** 10.1186/s12978-019-0784-8

**Published:** 2019-08-15

**Authors:** Ibrahim Yakubu, Gholamreza Garmaroudi, Roya Sadeghi, Azar Tol, Mir Saeed Yekaninejad, Adadow Yidana

**Affiliations:** 1 Department of Health Education And Promotion, School of Public Health Tehran University of Medical Sciences, Tehran, Iran; 2 Department of Nursing, Nursing and Midwifery Training College, Gushegu, Ghana; 3 Department of Epidemiology and Biostatistics, School of Public Health Tehran University of Medical Sciences, Tehran, Iran; 4 Department of Community Health and Family Medicine, School of Medicine, University for Development Studies, Tamale, Ghana

**Keywords:** Sexual Abstinence, Adolescent pregnancy, Prevention, Health belief model, Ghana

## Abstract

**Background:**

Adolescent pregnancy is a worldwide problem because of its health, social, economic and political repercussions on the globe. Even though the rates of adolescent pregnancy have declined over the decade, there is still unacceptably high rates especially in lower and middle-income countries including Ghana. Although the problem has been widely investigated, there is little information on the effectiveness of different methods to improve adolescent sexual abstinence based on theoretical models. This study is aimed to assess an educational intervention program on sexual abstinence based on the Health Belief Model (HBM) among adolescent girls in Northern Ghana.

**Methods:**

A cluster randomized control trial was conducted in Ghana from April to August 2018. Participants within the ages of 13–19 years were enrolled voluntarily from six randomly selected Senior High Schools (3 for intervention and 3 for control). A total of 363 adolescent were enrolled. A self-structured questionnaire was administered to both groups of participants at baseline and endpoint of the study. Control participants received their normal classes whiles the intervention group additionally received comprehensive sexuality education for 1 month. Qualified midwives conducted the health education program. At least two sessions were conducted for each participating class weekly. The lessons focused on perceived susceptibility, perceived severity of adolescent pregnancy, perceived benefits, perceived barriers to adolescent pregnancy prevention, personal and family values, perceived self-efficacy and knowledge of contraceptives. Educational strategies such as discussions, demonstrations, role-play and problem solving techniques were used to deliver the lessons. Sexual abstinence was the outcome variable of the study and it was measured after 3 months of the intervention. Binary logistic regression was used to assess the impact of the intervention on sexual abstinence practice.

**Results:**

At baseline, there was no difference between control and intervention groups. The mean score of Knowledge and attitude for control were (58.17 and 139.42) and intervention (60.49 and 141.36) respectively. Abstinence practice was 69.4% for control and 71.6% in the intervention group. However, after the intervention, the mean score of knowledge and attitude for control were (87.58 and 194.12) respectively. Sexual abstinence in the control was 84.4% and intervention was 97.3% respectively. The educational interventions resulted in a significant difference in sexual abstinence between intervention and control groups (OR = 13.89, 95% Confidence Interval (2.46–78.18, *P* < 0.003).

**Conclusion:**

Educational intervention, which was guided by HBM, significantly improved sexual abstinence and the knowledge of adolescents on pregnancy prevention among the intervention group. Provision of comprehensive sex education guided by behavioural theories to adolescents at Senior High Schools in Ghana is recommended.

**Trial registration:**

This trial was retrospectively registered in Protocol Registration and Results System (PRS) with trial number NCT03384251.

## Plain English summary

The rate of adolescent pregnancy has declined over the decade. However, there is still unacceptably high rates, especially in Ghana. Although the problem remains widely investigated, there is little information on the effectiveness of different methods to improve adolescent pregnancy prevention based on theoretical models in Ghana. This study aimed to assess an educational intervention program on knowledge, attitude and behaviour towards pregnancy prevention based on the Health Belief Model (HBM) amongst adolescent girls in Northern Ghana.

To assess the effectiveness of the educational intervention, an experimental study was conducted using a cluster randomized control trial in Ghana from April to August 2018. Adolescents between the ages of 13–19 years were enrolled voluntarily from six randomly selected senior high schools (3 in the intervention and 3 in the control groups). A total of 363 adolescents were enrolled. Research assistants administered the structured questionnaire to both groups of participants at baseline and endpoint of the study. Control participants received their normal classes whilst the intervention group additionally received comprehensive sexuality education from professional midwives for 1 month. Abstinence from sexual intercourse was the outcome variable of the study, which was assessed after 3 months of the intervention.

At the beginning of the study, there was no difference between control and intervention groups with respect to adolescents’ knowledge, attitude and sexual abstinence. However, after the educational intervention, there was increase in knowledge, change in attitude and improved practice of sexual abstinence among intervention group than the control group. The educational interventions resulted in a significant difference in sexual abstinence between intervention and control groups.

Educational intervention, which was guided by HBM, significantly improved the knowledge and sexual abstinence practice of adolescent towards pregnancy prevention among the intervention group. Provision of comprehensive sex education guided by behavioural theories to adolescents at Senior High Schools in Ghana is recommended.

## Background

Adolescent pregnancy is a complex phenomenon that affects families, health care services, education, governments, and the youths themselves. About 16 million girls aged 15 to 19 and some 1 million girls under 15 years give birth every year, mostly in low and middle-income countries [1]. A considerable percentage of adolescent pregnancy are planned in low and middle-income countries where several women still marry early. However, even intended pregnancies to undeveloped women in limited resource settings is a matter of public health concern because of the dangers associated with it. Complications during pregnancy and childbirth are the second cause of death for 15–19-year-old girls globally [1]. Evidence suggest that young adolescents are prone to experiencing obstructed labour, fistula, and premature childbirth and to deliver low birth weight babies than older women. Babies of adolescent mothers are at higher risk of dying than those of women aged 20 to 24 years [1, 2] because those children are at risk of malnutrition, low mental and physical development, inappropriate social connection with parents and poor education [3, 4].

There are numerous factors associated with adolescent pregnancies. These factors include early marriages [5], inadequate social and economic support [5, 6]. Curiosity and peer pressure [7, 8], poor sex education [9], insufficient reproductive health services [10, 11] and health workers unprofessionalism to providing contraceptive services for adolescents [8, 12]. In addition, unmet need for contraceptives by adolescents [13] and the myth of contraceptive side effects [7, 14] reduces contraception the uptake. Also, low-risk perceptions, poor skills of adolescents to negotiate safer sex options [15] as well as early sexual debut [16] have all contributed to adolescent pregnancy.

Ghana has implemented a number of policies to improve adolescent development through the provision of youth focus friendly health services in the country including the school health program [17]. However, the school health program limits sex education to providing bio-medical facts and warning of negative consequences about adolescent pregnancy and not relating it to adolescent’s sociocultural context [7, 15, 18]. In addition, most studies conducted in Ghana are descriptive in nature; identifying factors associated with adolescent pregnancy [7, 15, 18–20] but does not include studies that assess pragmatic interventional programs to prevent the occurrence of adolescent pregnancy through sexual abstinence. Interventional studies related to adolescents sexual abstinence towards pregnancy prevention lack theoretical model as guides. Health Belief Model (HBM) is recommended as a useful model to explain health behaviours including adolescents’ risky sexual behaviours [21]. Indeed the HBM is one of the appropriate health promotion models designed to predict preventive health behaviours [22–24], and it has enhanced preventive health behaviours in breast cancer screening and prevention of risky sexual behaviours in adolescents [25]. HBM is a theoretical model that has been constructed from six domains. These domains are perceived susceptibility, perceived severity, perceived barriers, perceived benefit, cue to action and perceived self-efficacy [21]. Based on the concept of HBM, adolescents must have some knowledge and motivation towards preventing pregnancy. They must perceive themselves as vulnerable to getting pregnant and they must be convinced that getting pregnant is a serious issue that has social, economic and health problems. Additionally, adolescents must be convinced that it is possible to obtain control over the social, economic and personal barriers and that the barriers do not outweigh the benefits of delaying pregnancy. Furthermore, an internal or external stimulus-cues to action-must trigger the health behavior of adolescents towards delaying pregnancy. Finally, adolescents’ must believe that they are able to delay getting pregnant (self-efficacy).

Schools are the best site for providing health education and promotion interventions because students spend most of the time in school and health promoters have the opportunity of reaching a large number of participants [26–28]. Students of Senior High Schools in Ghana are adolescents and most of them are sexually active and has demonstrated little knowledge and use of contraceptives [7, 15, 20, 29]. Abstinence based sex education encourage that sex be delayed until marriage and teaching of contraceptives is restricted to its negative effects. Evidence suggest that abstinence only programs on are not effective [30]. Therefore, to render comprehensive sex education to adolescents in schools has the potential to increase their knowledge; enhance their attitude and behaviour towards pregnancy prevention. This study was intended to evaluate the impact of an educational intervention program on the knowledge, attitude and behaviour towards pregnancy prevention based on Health Belief Model amongst adolescent girls in Northern Ghana.

## Methods and materials

### Description of study area

The study was conducted in the Tamale Metropolis, which is the fourth largest city in Ghana. It is the capital town of the Northern Region with a population of about 400 thousand [31]. The population of the metropolis is youthful (almost 36.4% of the population is below 15 years) depicting a broad base population pyramid which tapers off with a small number of elderly persons (60 years and older). An estimated 10,613 adolescents are distributed among the 14 (12 mixed and 2 single-sex) Senior High School within the metropolis [31]. Adolescent pregnancy rate in Ghana is 65 per 1000 adolescent but, that of northern region is 109 per 1000 [17]. As the capital of the Northern Region, students attending SHS in tamale are drawn from the various towns within the region, which is why it is appropriate to conduct the study in Tamale.

### Study design

This interventional study used Clustered Randomized Controlled Trial to assess the impact of an educational program on sexual abstinence among adolescent girls using a researcher-structured questionnaire based on the Health Belief Model. The study was conducted at six [6] selected Senior High Schools in northern Ghana. These schools were selected by random cluster sampling method from 12 mixed Senior High schools in the Tamale metropolis. The study was conducted on adolescent girls in selected Senior High Schools in Tamale, Northern Ghana. Only unmarried adolescent female high school students were enrolled in the study because we presume that they are not in any formal sexual commitment, therefore, they can delay sexual intercourse or practice safe sex. However, for lack of data on contraceptive use and practice of safe sex, we assessed sexual abstinence as the outcome variable of this study. Girls who do not want to participate were excluded from the study.

### Sampling

A multi-stage sampling method was used. Six [6] out of 12 mixed SHS were randomly sampled. We grouped the six schools into two clusters based on their locations. The two clusters were randomly allocated as an intervention and control group respectively. In each school, one class was selected by simple random sampling from each grade (SHS are in three grades) in both intervention and control groups. All adolescent participants in the class were considered a cluster and they were included in the study. The aim of this study was expounded to all potential participants in each selected classroom. All adolescent girls (between the ages of 13–19 years) in each selected classroom were enrolled voluntarily in the study.

**Fig. 1:**
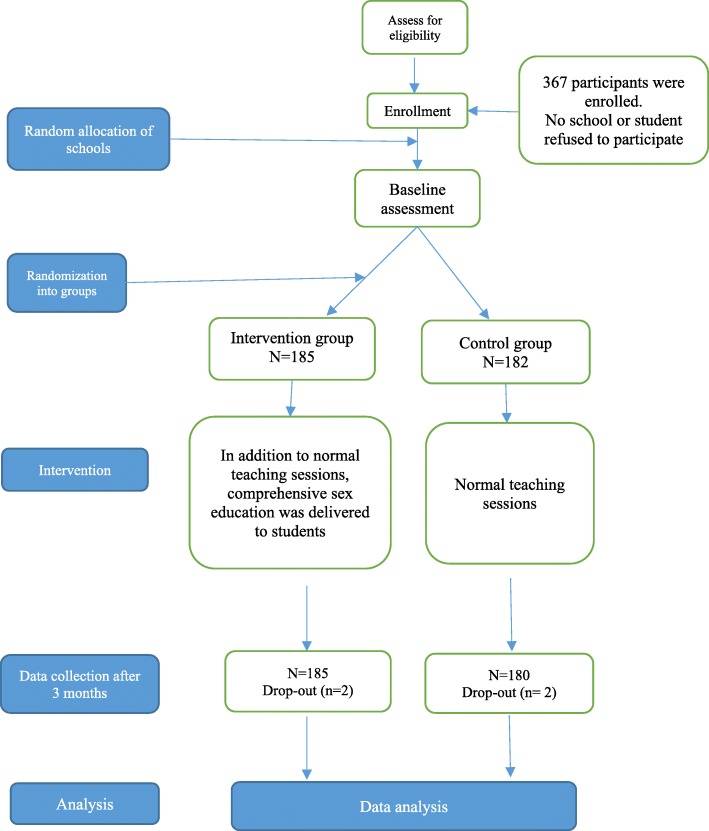
Clustered Randomized Control Trial flow chart based on consort reporting

A total of 367 (185 and 182) were enrolled in the intervention and control groups respectively. This sample size was reached by an estimation formula; calculating the mean score of sexual abstinence, knowledge and attitude of groups under study to detect five score difference between the two groups with 90% statistical power and 5% probability of type one [1] error. Consequently, 363 (183 = intervention and 180 = control) were used for analysis because four participants were lost to follow up. The study was conducted in accordance with the Consolidated Standards of Reporting Trials (CONSORT) guidelines [32] (Fig. 1).

**Fig. 2:**
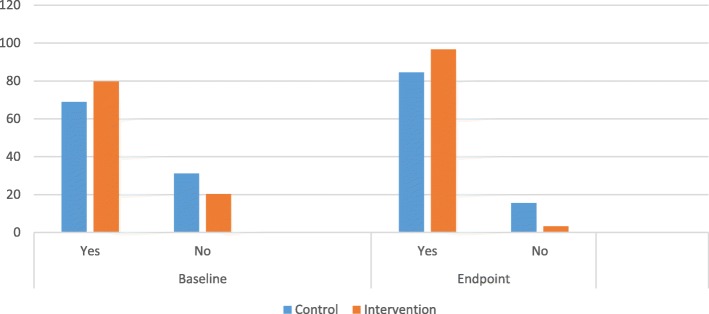
Abstinence at baseline and endpoint

### Variables

The dependent variables of the study was sexual abstinence (adolescents’ ability to delay/abstain from sexual intercourse) whilst the independent variables were knowledge of pregnancy prevention, the six HBM domains, age, social class, grade, ethnicity and birth order.

### Data collection instruments and measures

A standard questionnaire was designed by the researchers and used for this study. Expert evaluation of the standard structured questionnaires had an average Content Validity Index (CVI) of 0.85 and a reliability test of above 70%. Research assistants who doubled as instructors for the educational program administered the questionnaire. The questionnaires were administered to participants before the educational program and three months after the intervention.

A total of 58 items which were designed based on the HBM six domains were used to assess adolescents perception towards pregnancy prevention. The items specifically assessed adolescents’ opinion on their chances of getting pregnant; adolescents’ opinion on the seriousness of adolescent pregnancy and the consequences of getting pregnant; adolescents’ opinion on the importance delaying pregnancy, adolescents’ opinion on the barriers of delaying pregnancy; and adolescents’ confidence on their ability to delay pregnancy. The degree with which participants perceived each of these items were recorded using a five-point Likert’s scale ranging from “strongly disagree for a lower score to strongly agree for a higher score”. Each domain has the same minimum score of zero (0) and a maximum score of 40. All the scores for the HBM domain variables were expressed in percentages for analysis.

One question was used to assess abstinence practice. Abstinence practice was a binary variable where a score of one [1] was labelled “yes” and Zero (0) was labelled “no”. One question was used to assess participants’ intention to abstain from sexual intercourse. The question on intention was on a three-scale item; 1 = definitely will, 2 = not sure and 3 = definitely won’t. A score of one denotes low abstinence intention and a score of three denotes high abstinence intention. The intention score was expressed in percentages for analysis.

Ten questions were used to assess knowledge level of participants on the use of contraceptives and susceptibility of adolescent pregnancy. The responses to each question were recorded by “Yes” and “No”. One point was given for each correct answer and zero for an incorrect response. Therefore, each participant obtains a maximum knowledge score of 10 and a minimum of zero (0). Total score for each participants’ score was expressed in percentages.

### Intervention

A comprehensive sex education program was delivered in six [6] sessions comprising of lessons on susceptibility and severity of teenage pregnancy, personal and community values, female reproductive system, contraceptives and decision-making. Qualified midwives conducted the health education program. At least two sessions were conducted for each participating class weekly.

The first lesson focused on Perceived susceptibility and perceived severity. Educational strategies for the first lesson was lecturing and discussions. Statistics were provided for participants to appreciate the burden of the problem, then, through discussions, participants identified adolescents at risk of getting pregnant and the effects of adolescent pregnancy. The discussions were preceded by a poster presentation. The facilitators presented a pamphlet on adolescent pregnancy prevention.

The second lesson focused on perceived benefits and perceived barriers to adolescent pregnancy prevention. Some barriers discussed included: lack of sex education, inadequate parental counselling, poverty, broken-homes, Non-use of contraceptives, alcohol and substance abuse, lack of adolescent-friendly services at hospitals, the negative attitude of health workers towards providing reproductive health services for adolescents and peer pressure. Benefits elucidated for participants were: School completion, Enrolment into a tertiary institution, Job security, Improve the quality of life, deliver healthy babies, gaining physical and emotional maturity getting pregnant and being respected in the community. Educational strategies were brainstorming, discussions and role-playing (Appendix 1).

Third lesson focused on Personal and family values. Values were defined as the things adolescents support or against, the things people choose on their own, with no outside pressure, or the things people believe in and are willing to uphold. Adolescents were also taken through the effects of peer pressure, such as: to go against personal values, indulging in risky behaviours; disobey parents, truancy, drug and alcohol abuse etc. adolescents were guided to formulate their personal goals. The most featured goals were to abstain from sex, to become a professional, and to marry after tertiary education. Educational strategies were discussions and role-playing.

The fourth lesson focused on self-efficacy and knowledge of contraceptives. Lecturing demonstration and practice were the educational strategies employed. Various contraceptives were presented and a demonstration of how to put on the male and the female condoms were conducted using educational models. Some participants were guided to practice on the models. Lesson five focus on the female reproductive system. Educational materials were pictures and models whiles educational strategies was lecturing.

The final lesson was on self-efficacy (decision-making skills). Problem-based learning was conducted through simulation. Participants were grouped to discuss the scenario and to present their solutions. Our educational intervention was guided by attitudinal and behavioural intervention strategies adopted for the study based on the Taxonomy of Behavior Change Techniques (TBCT) for interventions [33] (Appendix 1) and Sharma (2011) educational process based on HBM constructs and behavioural objectives [34].

### Data analysis

Data was entered to Statistical Package for Social Science (SPSS) version 24. Baseline participant characteristics between intervention and control groups and sociodemographic variables were determined using descriptive statistics. Baseline knowledge level score and six HBM domains scores were compared between intervention and control groups.

Binary logistic regression was used to evaluate the impact of the educational intervention on participants’ sexual abstinence practice. The statistical significance level was determined at a 95% confidence interval.

## Results

### Participants’ characteristics

At baseline, approximately all participants’ characteristics were similarly distributed between intervention and control groups. One hundred and eighty (180) of the participants representing 49.6% of the study population were the control group whiles one hundred and eighty-three (183) that is 50.4% of the participants were in the intervention arm of the study. The participants who were between the ages of 14 to 16 years were 19.8% whilst 80.2% of them were between the ages of 17 to 19 years. The participants who were from a lower social class were 65, 32.2% were from middle class and 1.7% were from upper class respectfully. With respect to the grade of students, 160 of the adolescent girls representing 44.1% of the respondents were from Senior High School level one, 119 (32.8%) were from level two and 84 (23%) were from level three (Table 1). Out of the 363 participants, 204 (56.2%) came from households with ten [10] or fewer members, 106 (29.2%) came from households with members ranging from 11 to 20 and 53 (14.6%) were from homes consisting of members above 20. Table 1 outlines participants’ demographic characteristics.

**Table 1:** Study participants Characteristics (*N* = 363)

Characteristics	Categories	Combined frequencies *N* (%)	Control (*N* = 180) Frequency (%)	Intervention (*N* = 183) Frequency (%)
Group	Control	180 (49.6)		
	Intervention	183 (50.4)		
Age (years)	14–16	72 (19.8)	41 (22.8)	31 (16.9)
	17–19	291 (80.2)	139 (77.2)	152 (83.1)
Social class	Lower	239 (65.8	122 (67.8)	117 (63.9)
	Middle	116 (32.5)	54 (30.0)	64 (35.0)
	Upper	6 (1.7)	4 (2.2)	2 (1.1)
Grade	SHS One	160 (44.1)	74 (41.1)	86 (47.0)
	SHS Two	119 (32.8)	67 (37.2)	52 (28.4)
	SHS Three	84 (23.1)	39 (21.7)	45 (24.6)
Ethnicity	Dagombas	268 (73.5)	123 (68.3)	145 (79.2)
	Gonjas	31 (8.5)	23 (12.8)	8 (4.4)
	Ashantis	13 (3.6)	7 (3.9)	6 (3.3)
	Others	51 (14.1)	27 (15.0)	24 (13.1)
Religion	Islam	302 (83.2)	152 (84.4)	150 (82.0)
	Christianity	61 (16.8)	28 (15.6)	33 (18.0)
Birth order	1–5	309 (85.1)	153 (85.0)	156 (85.2)
	6–10	47 (12.9)	22 (12.2)	25 (13.70
	Above 10	7 (1.9)	5 (2.8)	2 (1.1)
Household Members	10 or less	204 (56.2)	96 (53.3)	108 (59.0)
	11–20	106 (29.2)	55 (30.6)	51 (27.9)
	Above 20	53 (14.6)	29 (16.1)	24 (13.1)

### Participants’ baseline and endpoint knowledge, abstinence from sex, intention to abstain from sex and HBM domains mean scores

At baseline, the mean Knowledge score was 58.17 and 62.28 for control and intervention group respectively. Three months after the intervention, the mean knowledge score was 60.49 and 87.58 for control and intervention group respectively. Adolescents mean attitude score towards pregnancy prevention at baseline was 139.42 and 141.36 for control and intervention group respectively. At endpoint, mean attitude score was 145.10 and 194.12 for control and intervention group respectively. Mean core of sexual abstinence at baseline was 71.35 and 74.89 for control and intervention group respectively. At endpoint, sexual abstinence mean score was 84.42 and 92.42 respectively for control and intervention groups (Fig. 2). Table 2 presents the mean score of knowledge, abstinence from sex, intention to abstain from sex and HBM domains.

**Table 2:** knowledge, abstinence from sex, intention to abstain from sex and HBM domains mean scores difference between intervention and control groups, adjusted for clusters at endpoint

Variable	Control (*N* = 180)		Intervention (*N* = 183)		P (ANCOVA)
	Before mean (SD)	After mean (SD)	Before mean (SD)	After mean (SD)	
Knowledge	58.17 (10.70)	62.28 (12.50)	60.49 (12.89)	87.58 (5.12)	<0.001
Perceived Susceptibility	43.92 (23.82)	42.72 (21.87)	47.13 (19.76)	95.71 (5.10)	<0.001
Perceived Severity	72.71 (23.08)	74.82 (21.66)	81.50(18.80)	95.93 (5.82)	<0.001
Perceived Barriers	67.89 (19.74)	67.79 (18.22)	69.40 (16.88)	84.23(13.84)	<0.001
Perceived Benefits	75.28 (21.33)	77.61 (20.71)	85.55 (14.87)	99.67 (1.12)	<0.001
Perceived Self-efficacy	61.86 (23.59)	63.54 (24.00)	75.89 (16.58)	83.74(14.64)	<0.001
Cues to action	44.93 (13.52)	44.86 (13.24)	43.31 (12.45)	43.36(12.75)	0.582
Attitude	139.42 (30.23)	141.36 (28.20)	145.10 (27.87)	194.12 (9.53)	<0.001
Intention to abstain from sex	84.44 (23.21)	93.33 (18.86)	89.89 (20.13)	98.45(5.22)	<0.001
Abstinence from sex	71.35 (27.21)	74.89(27.94)	84.42(22.76)	92.42(11.59)	0.001

At the end of the study period, data from 180 and 183 participants were collected from the control and intervention groups respectively. Two participants from each group were lost to follow up during the intervention period (Fig. 1). These four participants were from different cities and not available for endpoint data collection. Over 98 % of the participants were involved in the final analysis. The reason for the high participation rate was that participants were enrolled in schools and the general interest in the subject of this study amongst Senior High School students. Three months after the educational intervention, On ANCOVA analysis, knowledge, abstinence and HBM domain constructs of participants at endpoint revealed a statistically significant difference of all variable construct between intervention and control groups with the exception of cues to action (Table 2).

A binary logistic regression model was used to evaluate the impact of the educational intervention. To exclude possible interference, we inputted social class, age, knowledge, intention and HBM constructs at baseline as possible motivators of abstinence practice. The model was able to produce a significant difference between the intervention and control groups (OR = 13.89, CI = 2.46–78.18, *P* < 0.003). The model was also able to revealed that perceived severity (OR = 1.03,CI = 1.00–1.06, *P* < 0.034), knowledge (OR = 0.94, CI = 0.903–0.998, *P* < 0.028), age (OR = 1.56, CI = (0.99–2.46, *P* < 0.05) and intentions to abstain from sex (OR = 1.04, CI = 1.02–1.06, *P* < 0.0001) were motivators of abstinence practice (Table 3). The rest of the HBM constructs were not motivators of abstinence practice at *p*-value <0.05 (Table 3).

**Table 3:** Logistic regression model on sexual abstinence practice

Variable		OR (95% CI)	*P*
Study group	Control^a^	1.00	0.003
	Intervention	13.89(2.468–78.188)	
Social class	Low social Class^a^	1.00	0.901
	High	1.075 (0.347–3.325)	
Age of participants		1.56 (0.99–2.46)	0.051
Perceived Susceptibility		0.99 (0.99–2.468)	0.653
Knowledge		0.94 (0.90–0.99)	0.028
Perceived Severity		1.03 (1.00–1.06)	0.034
Perceived Barriers		0.98 (0.96–1.01)	0.384
Perceived Benefits		1.02 (0.99–1.05)	0.172
Perceived Self-efficacy		0.99 (0.96–1.03)	0.977
Cues to action		0.99 (0.96–1.03)	0.758
Intention to abstain		1.04 (1.02–1.06)	<0.001

^a^Set to zero because this parameter is redundant

## Discussions

Comprehensive sex education program provided to adolescents in SHS based on HBM significantly improved sexual abstinence in the intervention group. The logistic regressions model predicted that the intervention group were 13 times likely to abstain from sexual intercourse than their counterparts in the control group. The study also revealed that knowledge of adolescent pregnancy prevention, perceived severity and intentions to abstain from sex were motivators of abstinence practice. Three months after the educational intervention, participants in the intervention group showed improved knowledge and attitude towards adolescent pregnancy prevention. They perceived themselves susceptible to adolescent pregnancy and believed that the consequence of adolescent pregnancy was far-reaching. The cost and benefits of delaying pregnancy were perceived to be rewarding, therefore, adolescents in the intervention group showed more confidence in their ability to delay pregnancy by abstaining from sex.

Several interventions have been conducted with the aim of reducing adolescent pregnancy [35–37]. School-based sexuality training programs meant to reduce risks of teenage pregnancy largely encourage one of two kinds of information concerning sexual activity: abstinence-only messages, or comprehensive sexuality education messages. Abstinence based sex education encourage that sex be delayed until marriage and teaching of contraceptives is restricted to its negative effects. Evidence suggest that abstinence only programs are not effective. Comprehensive sexuality education programs include abstinence messages; nevertheless, it correspondingly deliver information on birth control techniques to prevent pregnancy [38–40]. Comprehensive sexuality education programs is suggested to delay adolescents’ initiation of sexual activity and by extension prevention of adolescent pregnancy [39]. Some of these programs used various methods such as the use of text messages [41] and parental support [42] to augment comprehensive sexuality education in order to reduce teenage pregnancy. Although some studies used social science theoretical model to guide their interventions, none of them used the HMB. That is why our intervention used HBM, targeting the knowledge and practice of sexual abstinence.

The goal of most adolescent pregnancy intervention strategies is either to delay sexual initiation or promote safe sex. Even though we conducted comprehensive sexuality education, there were inadequate data on contraceptive use by our participants. Therefore, our study was narrowed to adolescents’ sexual abstinence behaviour for a short-term (three months) outcome. Three months after the intervention, participants in the intervention group reported a reduction in sexual activity than their counterparts in the control group. Because this is a short-term measure, we cannot guarantee that the reduction in the sexual activity will continue in the long term. Nevertheless, a study conducted by Jemmott used the theory of planned behaviour in his intervention, which helped to reduce sexual behaviour [43]. This is consistent with our study where participants in the intervention group reported reduced sexual activity. Similarly, Chunyan et al. [44] in their study conducted in China found that students’ experience of school-based sexuality education was positively associated with their sexual and reproductive health knowledge, sexual behaviours and reproductive health outcomes. Furthermore, Villarruel et al. [45] reported that participants were less likely to initiate sexual intercourse during the intervention or follow up period than control among a mixed gender sample in their control trial experiment. Using peer-led sex education in a school-based randomized trial, Stephenson (2004) reported that adolescent reported less intercourse in the peer-led intervention group than in the control arm [46]. Contrary to our study, Roosa et al. [47] opined that abstinence interventions do not reduce sexual behaviour in the long term and that abstinence is not long-lasting. It is worth noting that the objective of intervention programs for adolescent pregnancy is to delay pregnancy and not to avoid it completely. Therefore, it is not out of place to conduct sexual abstinence intervention programs as we have done in this study.

Parental support for adolescents has also enhanced adolescent pregnancy prevention. This is demonstrated by a study conducted by Baku et al. [48] on the effects of training on parents’ knowledge and attitude towards adolescent sexuality; they found that training parents for a relatively short period could positively influence parents’ knowledge and attitudes about adolescent sexuality.

Research shows that a lack of knowledge about adolescent pregnancy prevention contributes to increasing rates of adolescent pregnancy [15, 49, 50] in Africa. It is therefore not surprising that after our intervention knowledge of adolescent pregnancy prevention motivated the practice of sexual abstinence. In other studies, knowledge has positively predicted abstinence from sexual intercourse among Wolaita Sodo University Students in Ethiopia [51]. Similarly, as reported by Gelibo et al. [54] also found that knowledge was a significant motivator of abstinence. In addition, Bazargan et al. [52] used information-motivation-behaviour-model to predict sexual behaviour in California, USA. They found that knowledge and perceived peer influence against sexual behaviour predicted sexual refusal skills. Therefore, for effective prevention of adolescent pregnancy, interventions should increase the knowledge of adolescents regarding adolescent pregnancy prevention methods.

Perceived severity of adolescent pregnancy was found to motivate adolescents to abstain from sexual intercourse. If adolescents get pregnant whiles in school, they are most likely not going to complete secondary education and they will be at risk of developing poor health conditions [1], suffer stigma and social isolation [53] as well as depression [54]. Therefore, the thought and experience of adolescents regarding adolescent pregnancy implications put fear in them hence, motivates the practice of sexual abstinence. All other things being equal, most adolescents will prefer not to get pregnant whiles in school.

One other important finding from this study is the motivation of adolescents to abstain from sexual intercourse by intention. Participants who intended to practice abstinence at the beginning of the study practised abstinence at the end of the study. Some other studies reported perceived behaviour control and positive attitudes to motivate adolescents intentions to use condoms [55], therefore, the motivation of abstinence by intentions is a build-up of previous studies that use intentions as an outcome variable. Intervention programs to prevent adolescent pregnancy should also aim at motivating adolescents to build the intention to delay pregnancy. Guiding adolescents to develop life goals and objectives will enable adolescents to take steps towards achieving the set goals.

As a strength of this study, a team of motivated health professionals who understood the subject matter conducted the study and members of the team delivered the sessions of their expertise. This improved the quality of the educational package. Another strength of the study was the high level of participation by the respondents. Furthermore, we were able to control information contamination and transfer between the intervention and the control groups by using a clustered randomized control trial. Finally, this study is one of the few studies, which actively recruits participants to assess the effect of an intervention towards adolescent pregnancy prevention in Ghana.

Social desirability bias is a severe limitation affacting a questionnaire-based approach for an outcome that could be considered socially desirable. This could affect accurate responses from participants. Even though the participants were comfortable and excited about the study, which is why we had almost 98% participation during the study, there was the possibility of inaccurate responses. The study was based on participants’ self-reported behaviour.

Cluster randomised trails are known for selection bias. We used probability-sampling method in this study and participants were identified before the random allocation of clusters. We anticipate that this will solve the issue of selection bias. Another limitation of this study is the issue of interdependence; participants from one school are more likely to be similar compared to other schools. This could affect the finding of the study. Furthermore, the timing for the educational intervention was challenging because it affected participating schools timetable and participants’ rest periods. The period of one month allocated for the intervention was not enough to cover all the classrooms since we had to conduct it in three classes for each school. For the study not to include male adolescents, adolescents out of school and married adolescents, findings from this study may not be representative and generalized for all adolescents in Ghana.

Replication of this study in other high-risk populations in Ghana would be beneficial to help develop policy guidelines. Future research should also include adolescent boys to determine whether gender has any effect on adolescent pregnancy prevention and the participants should be followed for at least six months. Furthermore, home-to-home intervention should be conducted to include adolescents out of school and their parents.

## Conclusion and recommendations

The comprehensive sexuality educational program, which was guided by HBM, reduced sexual activity of adolescents in the short-term and improved their knowledge on pregnancy prevention. Based on this study, we recommend that comprehensive sex education should be provided to adolescents at Senior High Schools in Ghana. Such educational intervention should focus on increasing adolescent s’ knowledge, perceived susceptibility and severity of adolescent pregnancy as well as enhancing adolescents’ intentions to abstain from sexual intercourse.

## Data Availability

The datasets used and/ or analysed during this study are available from the corresponding author on reasonable request.

## Appendix

**Table 4:** Intervention strategies for pregnancy prevention based on HBM

Target variable	Procedure	Practice techniques	Educational strategies
Perceived susceptibility and Perceived severity	General information about behavioural risk, including susceptibility to adolescent pregnancy, the severity of adolescent pregnancy	Provide information on behavioural risk through posters, pamphlets and discussions	Lecturing Discussion
Perceived benefits Perceived barriers	Information about the benefits and cost of getting pregnant, focusing on what will happen if a person does or does not get pregnant	Provide information on the significance of not getting pregnant as adolescents	Discussion Brainstorming Lecturing
Perceived barriers	Identify barriers to of avoiding pregnancy and ways of overcoming them	Identify barriers to abstinence	Problems based learning Role-playing Discussions
Perceived self-efficacy Knowledge of contraception	Information on contraceptives And the demonstration of how to put on condom	Provide instruction and model demonstration	Lecturing Demonstrations Practice
Knowledge of reproductive system and reproduction	Information on reproductive organs and their functions	Provide information through posters, pictures and models	Demonstration Lecturing
Perceived self-efficacy	Information on decision-making skills.	Encourage decision-making through role-playing and scenarios.	Problem-based learning

## Abbreviations

CONSORT: Consolidated Standards of Reporting Trials
CVI: Content Validity Index
HBM: Health Belief Model
SHS: Senior High School
SPSS: Statistical Package for Social Sciences
TBCT: Taxonomy of Behavior Change Techniques
WHO: World Health Organization
